# Supplementary material for: Mobility and ART retention among men in Malawi: a mixed‐methods study
Source: J Int AIDS Soc. 2023 Mar 21;26(3):e26066. doi: 10.1002/jia2.26066 (PMC10029992; doi:10.1002/jia2.26066)
Supplement: Supplementary file 1 — Appendix A: Socio‐demographics and ART history of IDI participants (n = 32). [file JIA2-26-e26066-s001.docx]

**Appendix A: Socio-demographics and ART history of IDI participants (n=32)**

| **Demographics** |  |
| --- | --- |
| Age, median years (IQR) | 39 (34-45) |
| Married, n (%) | 27 (84%) |
| **ART** **experience** |  |
| Years since diagnosis, median (IQR) | 1.7 (0.8-9.0) |
| Ever initiated ART, n (%) | 30 (94%) |
| Disclosed HIV status to primary partner | 26 of 27 with partners |
| **Socioeconomic status** |  |
| Household access to a cell phone, n (%) | 16 (50%) |
| Has savings, n (%) | 6 (19%) |
| Attended any secondary school, n (%) | 13 (41%) |
